# Effect of CNTs and GO Additives on Mechanical and Electrochemical Properties of Cement Structural Supercapacitors

**DOI:** 10.3390/ma19102116

**Published:** 2026-05-18

**Authors:** Yumin Zhang, Wenhao Zhao, Zizhu Fang, Senlin Li, Ye Wu, Kewei Sun, Longhai Feng, Zhicheng Yu, Jin Wang, Hao Yang

**Affiliations:** 1 College of Materials Science and Engineering, Nanjing Tech University, Nanjing 211816, China; 2 Materials and Structural Engineering Department, Nanjing Hydraulic Research Institute, Nanjing 210029, China

**Keywords:** supercapacitors, carbon nanotubes, graphene oxide, cement/carbon composites, electrochemical performance

## Abstract

This study presents a hierarchical conductive-network strategy to overcome the performance trade-off in cement structural supercapacitors (CSSCs). By incorporating one-dimensional carbon nanotubes (CNTs) and two-dimensional graphene oxide (GO) into Portland cement, we simultaneously enhance its electrochemical and mechanical properties. The approach exploits the complementary roles of the two nanomaterials: CNTs establish a three-dimensional percolation network that facilitates electron transport, while GO promotes formation of a denser calcium silicate hydrate (C-S-H) gel and refines the pore structure by complexing with calcium ions, thereby improving ionic pathways. The k12gc sample attains a specific capacitance of 66.8 F g^−1^ at 0.1 mA cm^−2^, a 58.4% rise in conductivity and a 63% reduction in charge-transfer resistance. At the same time, the composite reduces harmful macropores by 27.9% and strengthens the material, with compressive and flexural strengths increasing by 4.8% and 8.3%, respectively. This work establishes a rational design principle based on functional division between CNTs and GO for developing high-performance, multifunctional CSSCs.

## 1. Introduction

The global shift to renewable energy systems is a critical pathway to carbon neutrality, and its success depends on overcoming the inherent intermittency of renewable generation. Photovoltaic and wind technologies achieve high conversion efficiencies, but their stochastic output leads to an annual energy curtailment rate of approximately 15% in modern renewable power grids, creating an urgent need for advanced energy storage solutions that offer both scalability and operational efficiency [1].

Contemporary energy-storage technologies exhibit a basic trade-off: electrochemical batteries deliver high energy density but limited power output, while electrostatic capacitors enable rapid charge transfer at the cost of low energy storage. Supercapacitors occupy the intermediate regime, combining very high power density, millisecond-scale charge–discharge kinetics, and exceptional cycle stability (>100,000 cycles) [1]. These attributes make them well-suited to applications such as frequency regulation and regenerative braking, where managing power pulses is more important than storing large amounts of energy.

Structural supercapacitors (SSCs) constitute a paradigm-shifting innovation that transcends conventional design limits by integrating multiple functions [2,3,4]. A typical SSC architecture comprises two principal components: structural electrodes and a structural electrolyte/separator system.

Current fabrication strategies for structural electrodes pair mechanically robust substrates with porous carbon materials. Wang et al. produced a brick-structured electrode by radical polymerization of PEDOT nanofibres on ceramic substrates, achieving a high specific capacitance (11.5 kF m^−2^) but with limited scalability [5]. Muralidharan et al. created CNT-decorated stainless steel mesh electrodes combined with Kevlar–glass fibre separators, which exhibited exceptional mechanical strength (>5 GPa) but low energy density (3 mWh kg^−1^) [6]. Reece et al. fabricated honeycomb sandwich structures with aluminium cores and reported promising specific capacitance (153 F g^−1^ at 0.6 mA cm^−2^), while noting that most designs confine active materials to surface regions and thus severely limit volumetric energy storage capacity [7].

Integrating energy storage into load-bearing materials, as exemplified by cement-based structural supercapacitors (CSSCs), offers a transformative path for sustainable infrastructure. Early proof-of-concept studies established the principle by creating percolating conductive networks in cement via carbon additives. The seminal work by Chanut et al. [8] showed that adding nano-carbon black (nCB) produced composites with measurable energy storage (200–220 Wh m^−3^) while preserving structural integrity, thereby validating the basic feasibility of CSSCs.

Subsequent research has followed two parallel paths to improve performance: optimising the cement-based electrode (CCE) and developing the cement-based solid electrolyte/separator (CBE). For electrodes, a persistent intrinsic trade-off remains: approaches that increase capacitance—such as raising the conductive carbon content—tend to weaken mechanical strength by disrupting cement hydration and increasing porosity.

Recent advances in carbon–cement electrodes (CCEs) have emphasised microstructural engineering and hybrid filler architectures. Yan et al. [9] showed that precise control of pore structure and carbon distribution can raise areal capacitance to 2188 mF cm^−2^, although compressive strength falls to 5.51 MPa at high carbon loadings. Mao et al. [10] systematically compared CNT and CF as conductive additives in CB–cement electrodes and found that 1.5 wt% CNTs form denser, more continuous electron-transport pathways via tunnelling effects, yielding an areal capacitance of 1562 mF cm^−2^ and a compressive strength of 23.1 MPa. Li et al. [11] used CF as conductive bridges between nCB agglomerates and demonstrated that 2 vol% CF combined with 5% nCB delivers an energy density comparable to that of 15% nCB alone (30.26 μWh cm^−3^) while maintaining 36.88 MPa compressive strength. Guo et al. [12] translated the CCE concept to practical mortar by designing a tri-network that combines CNT-reinforced cement paste with rGO-coated conductive aggregates, achieving 350.0 mF cm^−3^ volumetric capacitance and retaining 32.22 MPa compressive strength. Most recently, Liu et al. [13] engineered a multiscale hydrogel network (MSHN) inside carbon–cement electrodes by SDS-mediated in situ polymerization of PAM around CB surfaces, producing an areal capacitance of 1708 mF cm^−2^, >83% retention after 10,000 cycles, and robust performance under mechanical loading, fire exposure, and across −20 to 80 °C.

Significant progress has been made concurrently in developing cement-based solid electrolytes. Early studies employed composites such as metakaolin and polyacrylic acid–cement to achieve favourable ionic conductivity and mechanical strength [14,15,16,17,18,19]. More recent advances include polymer–cement hybrids and bioinspired laminated architectures. Zhang et al. [20] produced dual PAM-network cement electrolytes with ionic conductivity of 68.54 mS cm^−1^ and compressive strength of 47.96 MPa. Shi et al. [21] reported in situ-polymerized PAAS–cement electrolytes that simultaneously improved conductivity (26.7 mS cm^−1^) and strength (44.7 MPa). Lin et al. [22] fabricated ice-templated, lamellar cement–PVA solid-state electrolytes with ionic conductivity of 27.8 mS cm^−1^ and compressive strength of 26.5 MPa. Xing et al. [23] achieved superionic behaviour (~101 mS cm^−1^) in oriented, layered magnesia–cement/PAM electrolytes. Shi et al. [24] introduced redox-mediated porous cement electrolytes using KI, reporting 20.5 mS cm^−1^ ionic conductivity and 21.6 Wh kg^−1^ energy density. Oumer et al. [25] developed porous polymer–cement electrolytes incorporating K_3_[Fe(CN)_6_] as a redox mediator, and Zhao et al. [26] produced highly interconnected porous cement electrolytes with 42% porosity and ionic conductivity of 15.6 mS cm^−1^.

However, these advanced CBE components must often be paired with costly electrodes (e.g., nickel foam, reduced graphene oxide), which raises system cost and hinders scalability. A persistent bottleneck is the electrode–electrolyte interface: weak interfacial bonding and mismatched properties promote delamination and increase impedance under combined mechanical and electrochemical stress, undermining long-term durability [27,28].

Despite these advances, a key gap persists: most CCE enhancement strategies boost electronic conductivity but compromise mechanical integrity, while CBE strategies target ionic transport without supporting electronic percolation in the electrode bulk [29]. Few approaches address both electronic and ionic transport within a single electrode system, and even fewer do so at industrially viable, low additive concentrations.

To address these intertwined challenges, this study proposes a targeted multi-scale engineering strategy. We incorporate a synergistic blend of one-dimensional carboxyl-functionalized CNTs (0.1 wt%) and two-dimensional GO (0.025 wt%) into an optimised NCB cement matrix. We hypothesise that the CNTs and GO provide distinct yet complementary functions: the CNTs form a robust three-dimensional percolation network to facilitate electron transport, while the GO templates a denser C-S-H gel and refines pore structure through complexation with calcium ions, thereby optimising ionic pathways and improving mechanical cohesion. This “electron–ion” functional partitioning within a hierarchical composite seeks to overcome the conventional performance trade-off by concurrently enhancing charge-transfer kinetics and mechanical reinforcement at the nanoscale, thus offering a route to high-performance CSSCs.

## 2. Experimental Methods

### 2.1. Materials

Ordinary Portland cement (P.O 42.5 grade) was provided by Hu Cheng 97 Building Materials Co., Ltd., Weifang, China. Its chemical composition is given in Table 1. Three carbon-based additives were employed: (1) Ketjen Black ECP-600JD nano-carbon black (NCB, Lion Corporation, Tokyo, Japan) with a specific surface area of 1270 m^2^ g^−1^; (2) carboxyl-functionalised multiwalled carbon nanotubes (CNTs, Suzhou Tanfeng Graphene Technology Co., Ltd., Suzhou, China) with a surface carboxyl group density greater than 1 mmol g^−1^; and (3) graphene oxide dispersion (GO, Suzhou Tanfeng Graphene Tech., Suzhou, China) containing 10 mg mL^−1^ monolayer sheets. A polycarboxylate superplasticiser was used to adjust workability. The mix proportions of all samples are listed in Table 2.

### 2.2. Preparation of Electrodes and Assembly of Supercapacitors

The wet mixture was prepared by a three-step dispersion protocol to ensure homogeneous nanoadditives. First, GO dispersion and CNTs were pre-dispersed by ultrasonication in an ice-water bath (0.5 W cm^−3^, 5 min). Next, NCB was added and the suspension subjected to high-speed shearing (20,000 rpm, 4 min). Finally, a deep ultrasonication treatment (40 kHz, 20 min) was applied to obtain a three-dimensional uniform distribution of the nanomaterials prior to casting.

The wet mixture was then gradually added to the dry components (cement and superplasticizer) in the planetary paste mixer together with 0.02 mol KOH aqueous solution [30]. Homogenisation proceeded through three successive cycles, each consisting of slow stirring at 145 ± 5 rpm for 1 min followed by high-speed mixing at 285 ± 5 rpm for 30 s.

Before casting, the PVC mould (200 mm × 150 mm × 1 mm) received surface treatment: a silicone-based lubricant was applied to the base plate and an oxygen-barrier polyethylene (PET) film was overlaid. The slurry underwent vibratory compaction and was levelled with a stainless-steel doctor blade. To prevent moisture loss, the cast specimens were immediately sealed with a bilayer film and cured at 20 ± 0.5 °C and 95 ± 2% relative humidity for 72 h. After demoulding, the slabs were punched into 22 mm diameter discs with a hydraulic punching apparatus and subjected to further curing under the same conditions until the specified testing ages. To produce uniform electrode surfaces, the discs were polished with 10,000-grit silicon carbide paper until mirror-smooth. Following thorough rinsing with ultrapure water to remove particulates, the electrodes were immersed in anhydrous ethanol for 24 h to halt hydration. They were then dried in a vacuum oven at 60 °C for 6 h, after which thickness was measured with a vernier calliper and mass with an electronic balance. The cement-based electrode preparation is illustrated in Figure S1.

Electrodes were immersed in 1 mol L^−1^ KOH at 25 ± 0.5 °C for 48 h to achieve electrolyte saturation. The cell assembly in a Swagelok test cell (23 mm diameter) involved the following sequence: a stainless-steel current collector, carbon paper pre-soaked in 1 mol L^−1^ KOH for 24 h (22 mm diameter), electrode, separator, counter electrode, carbon paper, and current collector. Prior to the final tightening of the cement-based structural supercapacitor, 500 μL of KOH electrolyte was introduced at each interface. The Swagelok cell was finger-tightened to a torque of around 0.5 N·m, resulting in a compressive stress of approximately 0.15 MPa on the electrode stack. The assembled cement-based capacitor is depicted in Figure S2.

### 2.3. Characterisation Tests

#### 2.3.1. Phase and Thermodynamic Analysis

We systematically examined the microstructural evolution and phase composition of CSSCs using advanced characterisation techniques. Scanning electron microscopy (SEM; Quanta FEG 250, Thermo Fisher Scientific, Hillsboro, OR, USA) at 15 kV was used to assess morphology and interfacial bonding. Phase identification employed X-ray diffraction (XRD; Cu-Kα radiation, 2θ range 20–80°) together with thermogravimetric analysis (TGA; heating rate 2 °C min^−1^ under N_2_) to determine crystalline structure and thermal stability. Mercury intrusion porosimetry (MIP) over 0.1–400 MPa quantified pore-structure changes resulting from the addition of NCB (12 wt%), GO (0.1 wt%) and CNTs (0.025 wt%).

#### 2.3.2. Mechanical Properties Testing

The mixed slurry was cast into a standard prismatic mould (40 mm × 40 mm × 160 mm) and compacted on a vibration table for 120 s to ensure adequate densification. Specimens were then cured under controlled conditions (20 ± 2 °C, RH ≥ 95%) for 28 days in accordance with ISO 1920-3:2019 [31]. Before mechanical testing, specimens were equilibrated at laboratory ambient (23 ± 1 °C, 50 ± 5% RH) for 24 h to minimise thermal-stress effects.

Compressive and three-point flexural tests were carried out on a servo-hydraulic testing machine (CDT1305-2; MTS Systems China Co., Ltd., Shanghai, China). Compressive strength was determined under displacement control at 0.6 mm min^−1^ (equivalent to a stress rate of 5 kN s^−1^ for standard specimens). Flexural tests used a crosshead speed of 0.2 mm min^−1^ (corresponding to a loading rate of 50 N s^−1^). Failure was defined as either visible macro-crack formation or a load drop exceeding 20% of the peak value, whichever occurred first. Three replicate specimens were tested for each condition.

#### 2.3.3. Electrochemical Testing

Electrochemical impedance spectroscopy (EIS) was carried out using a Bio-Logic Xworkstation(Bio-Logic SAS, Seyssinet-Pariset, France) with an AC amplitude of 5 mV and a frequency range of 0.01 Hz to 100 kHz. The electrode effective conductivity (σ_eff_) was then calculated according to formula 1 [32]. Cyclic voltammetry (CV) measurements were performed over a potential window of 0–1 V at scan rates of 5, 10, 15, 20, 50, 100, 200 and 500 mV s^−1^. A galvanostatic charge–discharge (GCD) test was subsequently recorded on a Neware (GYG-102797, Neware Technology Limited, Shenzhen, China) instrument between 0 V and 1 V at current densities of 0.1, 0.2 and 0.5 mA cm^−2^. From the charge–discharge curves, the specific capacitance (C_m_, F g^−1^), energy density (E, Wh kg^−1^) and power density (P, W kg^−1^) were computed using Equations (2)–(4) [33,34,35]. In all cases, specific capacitance, energy density and power density are reported per gram (g^−1^) of incorporated NCB.

σ_eff_ = L/(SR_s_) (1)

C = (i_m_ × ∆t)/∆V (2)

E = (C_m_∆V^2^)/7.2 (3)

P = (3600 × E)/∆t(4)

Here, L denotes the true separation between the two blocking electrodes (for example, stainless steel), R_s_ is the internal resistance of the CSSCs, i_m_ is the current density (A g^−1^), ∆t is the discharge time (s), i is the current, and ∆V is the voltage difference (V). In this context, ∆V is calculated by subtracting the initial voltage drop from the operating voltage range.

## 3. Results and Discussion

### 3.1. Phase and Thermodynamic Analysis

#### 3.1.1. Morphology

Scanning electron microscopy revealed distinct microstructural evolution across the composite systems. As shown in Figure S3a, the CNTs formed pronounced fibrous entanglements and agglomerates. This morphology reflects synergistic electrostatic and van der Waals interactions among their functionalised surfaces and underscores the inherent dispersion challenge [36]. Figure S3b shows the characteristic lamellar stacking of GO nanosheets.

**Figure 1:**
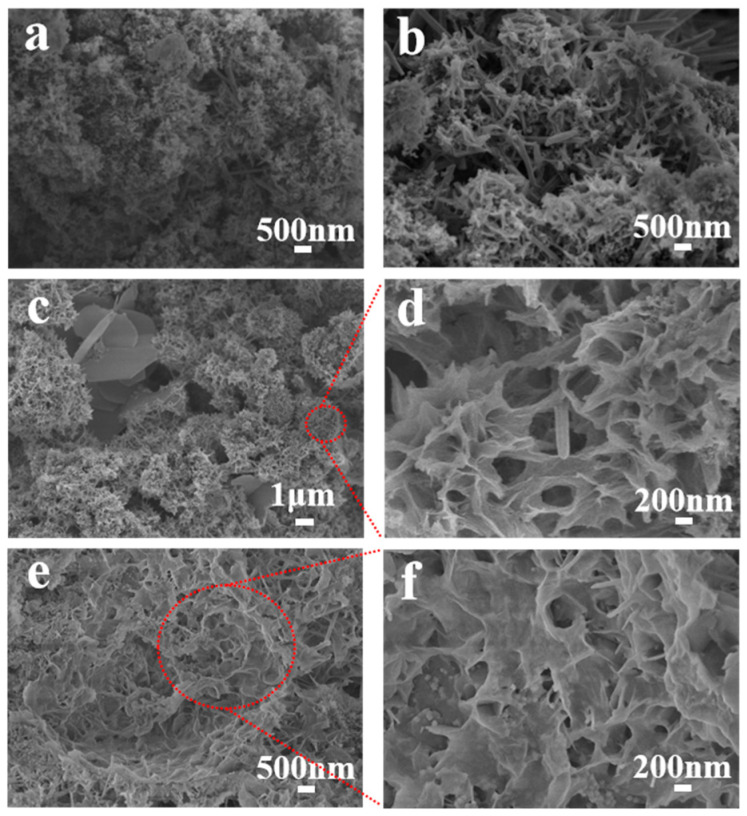
SEM images of each sample: (**a**) k12, (**b**) k12c, (**c**) low-magnification and (**d**) high-resolution image of k12g, (**e**) low-magnification and (**f**) high-resolution image of k12gc.

Figure 1 presents SEM images of the modified samples, and a comparative microstructural analysis reveals distinct evolutionary pathways. The cement-based electrode without nano-enhancement (k12, Figure 1a) shows a disordered microstructure in which AFt crystals are randomly dispersed throughout a loosely packed C–S–H gel. The specimen containing only CNTs (k12c, Figure 1b) likewise retains a chaotic distribution of hydration products. By contrast, the GO-only modified material (k12g, Figure 1c,d) exhibits a self-organising behaviour, producing uniformly sized, spatially ordered flower-like C-S-H crystals, although secondary agglomerates of NCB remain. Notably, the GO/CNT composite system (k12gc, Figure 1e,f) substantially refines the microstructure: GO sheets guide the development of a densely packed floral C-S-H gel, and primary NCB particles become homogeneously embedded in the gel network, with agglomerate sizes markedly reduced relative to the control.

The observed microstructural evolution stems from the multifunctional roles of GO. First, the oxygen-containing functional groups on GO complex with Ca^2+^ in the pore solution, thereby modulating local ion concentration and hydration kinetics [37]. Second, GO sheets act as heterogeneous nucleation sites that template the oriented growth of C-S-H gels into ordered morphologies. The two-dimensional GO also provides anchoring points that, through π – π interactions, inhibit NCB particle agglomeration and promote homogeneous dispersion [38,39]. Together with the percolation network formed by CNTs, these synergistic mechanisms regulate the composite microstructure and produce a densified matrix with uniformly distributed hydration products.

#### 3.1.2. X-Ray Diffraction Analysis and Thermogravimetric Analysis

**Figure 2:**
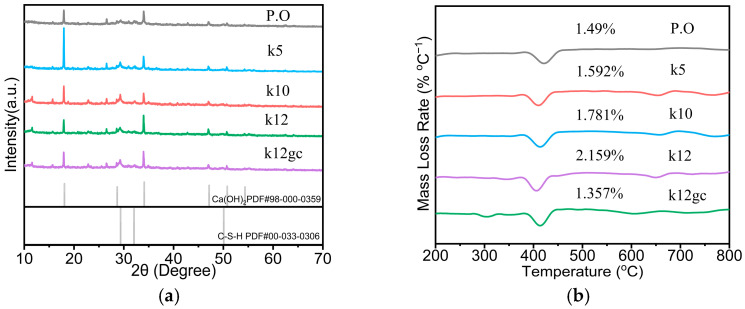
XRD and TG curves of various samples (**a**) XRD curve, (**b**) TGA curve.

Figure 2a presents XRD patterns of the composite samples. The peaks at 18° and 34° correspond to calcium hydroxide (CH) in the cement matrix, while the peak at 29° indicates calcium silicate hydrate (C-S-H) formed during hydration [40]. A comparison of the k12 and k12gc patterns shows identical peak positions, indicating that NCB, CNTs and GO do not chemically participate in the hydration reactions. Thus, the phase composition remains unchanged across specimens; CNTs and GO instead affect the microstructural arrangement of the hydration products, altering morphology by physical interaction with the matrix rather than by changing chemical identity.

Figure 2b shows the thermogravimetric (TG) curves of the various samples; the peak at approximately 410 °C corresponds to the decomposition of calcium hydroxide [41]. Comparison of the calcium hydroxide mass loss among the samples indicates a slight increase in CH content as the NCB fraction rises. The sample containing both CNTs and GO (k12gc) exhibits a marked reduction in CH content, an effect primarily attributed to the presence of GO. Although GO does not participate chemically in cement hydration, its addition alters the crystallisation and morphology of calcium hydroxide in the cement. Owing to its hexagonal lattice, GO hinders the formation of hexagonal plate-like calcium hydroxide crystals and refines their size [37,42].

#### 3.1.3. Mechanical Performance Testing

**Figure 3:**
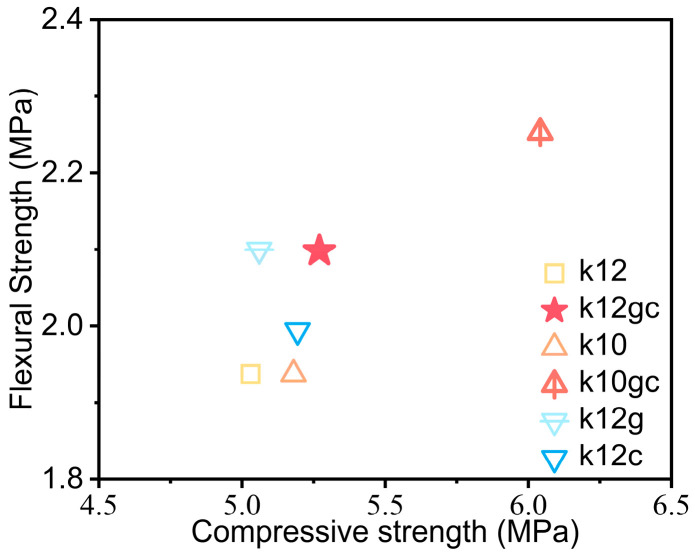
Compressive and flexural values of the samples.

Figure 3 shows the composites’ compressive and flexural strengths. Relative to the reference group (k10), the k10gc composite (10 wt% NCB, 0.1 wt% CNTs and 0.025 wt% GO) reached 6.04 MPa and 2.25 MPa, increases of 16.6% and 16.3%, respectively. The k12gc sample recorded smaller gains, at 5.27 MPa and 2.10 MPa, equivalent to improvements of 4.8% and 8.3%. Compared with systems modified by a single nanomaterial, the GO-CNT hybrid reinforcement shows a clear synergistic effect. These findings confirm that adding 0.1 wt% CNTs together with 0.025 wt% GO efficiently enhances the mechanical performance of the cementitious electrodes.

The improvement in mechanical properties arises from the synergistic, multi-scale reinforcement provided by GO and CNTs. First, carboxylated CNTs chemically adsorb onto active sites of C-S-H gels via their surface functional groups, creating strong interfacial bonds. Their high aspect ratio (>1000) allows them to bridge microcracks, thereby transferring stress and inhibiting crack propagation [43,44]. Second, GO nanosheets act as templates that guide the orderly growth of hydration products and thus reduce porosity. Their two-dimensional structure supplies heterogeneous nucleation sites for C-S-H gels, promoting the formation of uniformly sized, layered assemblies [45,46]. Third, synergistic dispersion of CNTs and GO diminishes the size of secondary NCB agglomerates to about 40% of their original value, which removes local stress concentration points. Together, these mechanisms—chemical bonding, physical reinforcement and structural regulation—furnish a theoretical basis for designing high-performance structural–functional integrated electrodes [9,14,47].

#### 3.1.4. Mercury Intrusion Porosimetry (MIP) Analysis

**Figure 4:**
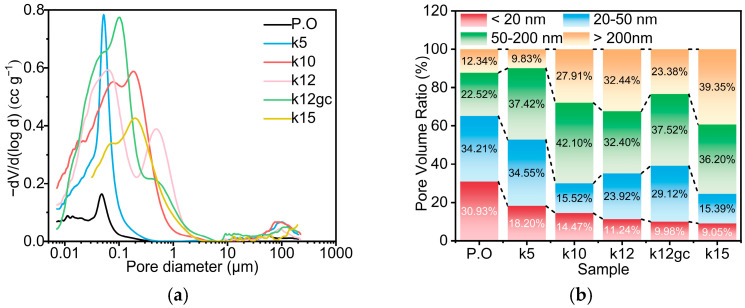
Porosity test results of each sample (**a**) Pore size distribution of each sample, (**b**) Distribution of harmful and harmless pores in each sample.

Figure 4 presents a mercury intrusion porosimetry (MIP) analysis that elucidates how nanoscale additives refine the microstructure. The pore size distribution (Figure 4a) indicates that adding 12 wt% NCB alters the porosity profile. Co-modification with GO and CNTs further optimises this distribution, shifting the pore population towards finer sizes.

To quantify the effect on mechanical durability, pores were classified according to the strength–pore structure relationship theory (Figure 4b). This classification divides pores into harmless (200 nm) categories. The addition of NCB alone increased the fraction of larger, detrimental pores, whereas the incorporation of GO and CNTs reversed that trend [48]. In the k12gc composite, for example, the proportion of highly harmful pores (>200 nm) declined by 27.9%, while the share of less harmful pores (20–50 nm) rose by 21.7%. This shift from larger, defect-inducing pores to smaller, less detrimental ones plausibly underpins the observed mechanical improvement.

Importantly, the same evolution in pore structure also improves ionic transport kinetics. The marked reduction in large, isolated pores (>200 nm) lowers the tortuosity of electrolyte infiltration paths and so facilitates faster ion diffusion through the electrode bulk. At the same time, the greater population of interconnected mesopores (20–50 nm) increases the volume of pores whose dimensions favour rapid formation and accessibility of the electrochemical double layer, thereby expanding the electrochemically active surface area available for charge storage.

The refined pore structure arises from the synergistic action of CNTs and GO. One-dimensional CNTs serve as nanoscale fillers and bridges within the matrix, subdividing larger voids into smaller, interconnected channels. Concurrently, two-dimensional GO sheets control hydration-product growth by a templating effect, encouraging formation of a denser, more integrated C-S-H gel network and thus reducing the volume of harmful pores. Together, the physical pore-filling and chemical microstructural templating produce a reinforced composite architecture with improved mechanical performance.

### 3.2. Electrochemical Testing

#### 3.2.1. Electrochemical Impedance Spectroscopy (EIS) Analysis

**Figure 5:**
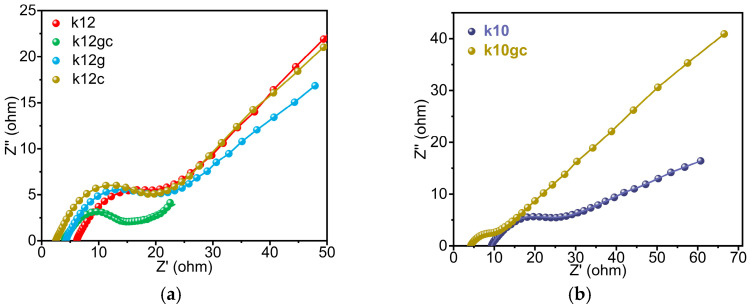
EIS spectra of various samples with different contents of NCB and modified treatment (**a**) 12 wt% of NCB, (**b**) 10 wt% of NCB.

**Figure 6:**
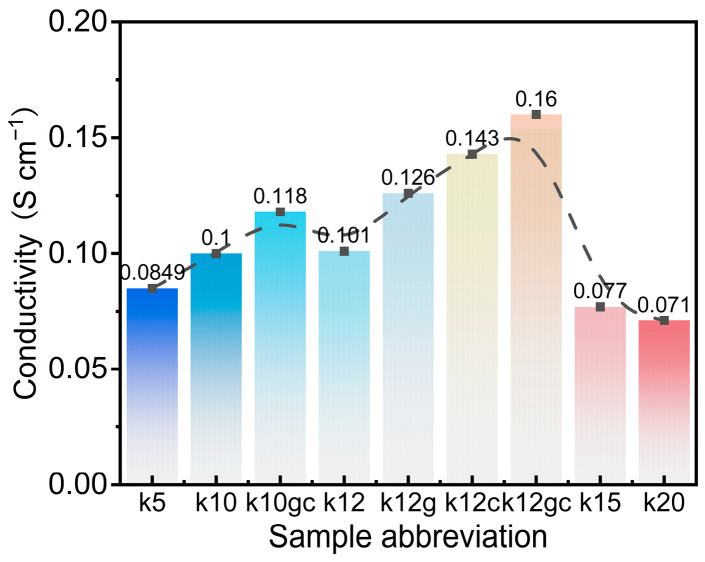
The effective conductivity of samples.

Electrochemical impedance spectroscopy and effective conductivity measurements provide direct evidence of improved charge-transfer within the cementitious electrodes (Figure 5 and Figure 6). A continuous three-dimensional conductive network forms owing to the homogeneous distribution of CNTs and GO nanosheets. In this network, CNTs act as electronic pathways and GO promotes ionic transport. This is illustrated by the k12gc composite, which shows the lowest equivalent series resistance (Rs = 4.28 Ω) and charge-transfer resistance (Rct = 13.0 Ω), together with a maximum effective-conductivity increase of 58.4% (Figure 5a and Figure 6). k12gc outperforms samples containing only CNTs (k12c) or only GO (k12g), indicating a synergistic interaction rather than a merely additive effect. Even at a lower conductive-agent loading (k10gc), the combined addition of GO and CNTs lowers Rct by 63% relative to the baseline sample without these nanoadditives (k10) (Figure 5b).

The improvement stems from the complementary roles of the two nanomaterials. The EIS results corroborate the functional division outlined in the Introduction: CNTs form an electronic-percolation backbone that bridges isolated NCB particles and lowers the bulk resistance, while GO facilitates ionic transport and interfacial charge transfer by templating the cement microstructure. Together they produce a synergistic reduction in both Rs and Rct, consistent with the 58.4% increase in conductivity observed for k12gc—an enhancement unattainable with either nanomaterial alone [49,50,51].

Exceeding the optimal NCB content (12 wt%) produced a 23.8–29.7% decline in effective conductivity (Figure 6), which is attributable to pronounced agglomeration that disrupts the conductive network. The superior performance of the co-doped sample k12gc (58.4% improvement) arises from a hierarchical conductive architecture. One-dimensional CNTs bridge isolated NCB particles and form a three-dimensional percolation skeleton that facilitates rapid electron transfer. At the same time, two-dimensional GO sheets, through their surface functional groups, establish organised ionic migration channels within the pore network. This complementary “electron highway–ionic channel” division of labour yields a synergistic, non-linear enhancement that exceeds the individual improvements produced by GO (41.6%) or CNTs (21.3%) alone. The modest gain observed in the 10 wt% NCB system further emphasises the existence of a percolation threshold, below which a continuous conductive network cannot be fully established.

Comparative experiments show that the 10 wt% NCB composite exhibits only a limited improvement in effective conductivity. This limitation stems from insufficient pore connectivity within the matrix, which prevents formation of a continuous conductive network. The observation therefore conforms to the universal percolation-threshold principle for conductive networks. Only when the NCB content reaches the critical value of 12 wt% does the spatial distribution of the nanomaterial align optimally with the matrix pore structure, allowing the full synergistic enhancement to emerge.

It is important to highlight that, although the relative enhancement in conductivity (58.4%) is considerable from a mechanistic perspective, the absolute effective conductivity (~0.16 S cm^−1^) is still significantly lower than that of traditional carbon-based electrodes. For applications requiring high power density, such as regenerative braking, further enhancements in absolute conductivity—achievable through optimisation of the conductive filler content and its dispersion, or by exploring alternative current collector designs—are essential. This study establishes the principle of functional division; however, applying this principle to achieve higher absolute performance levels warrants further investigation.

#### 3.2.2. Cyclic Voltammetry (CV) Test

**Figure 7:**
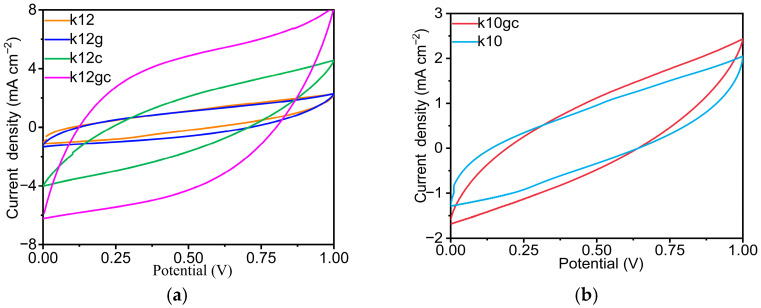
CV curves at a scan rate of 5mV s^−1^, (**a**) k12, k12g, k12c and k12gc (**b**) k10 and k10gc.

We systematically evaluated the electrochemical behaviour of cement-based supercapacitors by cyclic voltammetry. The CV curves of the k12gc sample recorded at 5 mV s^−1^ display a quasi-rectangular profile (Figure 7a and Figure S4), indicating dominant electric double-layer capacitance in the absence of discernible redox peaks. Quantitatively, the GO + CNTs co-doped specimen shows the largest integral area, 2.83, which exceeds the k12 reference by 118.4%. This improvement aligns with synergistic effects between CNTs, which provide high effective conductivity, and GO, which exerts regulatory effects; EIS measurements further support this interpretation by showing a reduced charge-transfer resistance. Comparable trends are evident for k10 and k10gc (Figure 7b and Figure S5). During charge–discharge, ions in the electrolyte must traverse the electrode surface rapidly [52,53]. If ions are not distributed uniformly or do not penetrate the electrode structure effectively, the effective capacitance falls and the voltage drop increases; this outcome arises primarily from the intrinsic electrical insulation of the cement matrix. Introducing NCB additives mitigates this limitation by creating ion-permeation pathways that enhance bulk conductivity.

#### 3.2.3. Galvanostatic Charge–Discharge (GCD) Test and Long-Term Cycling Test

**Figure 8:**
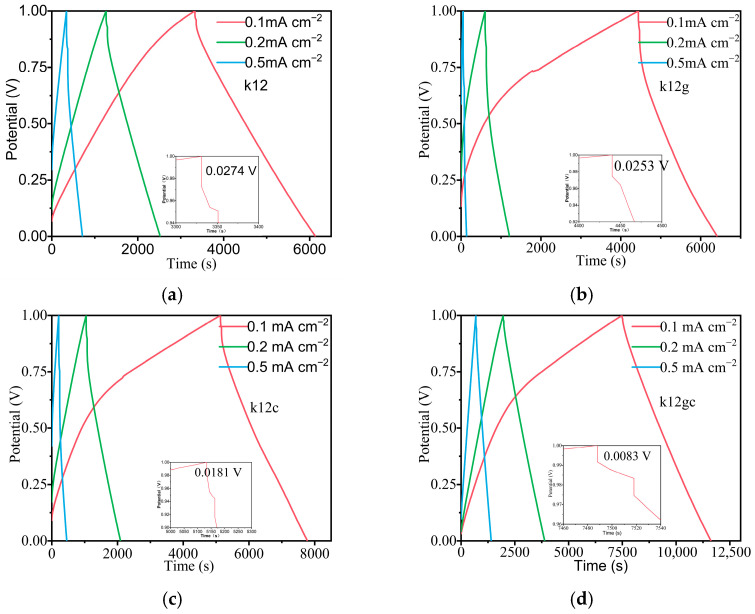

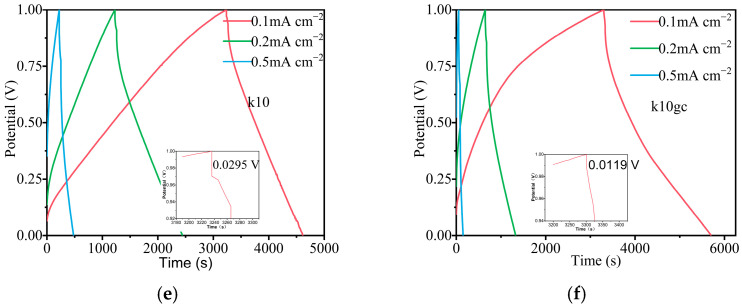
GCD curves of the sample (**a**) k12, (**b**) k12c, (**c**) k12g, (**d**) k12gc, (**e**) k10, (**f**) k10gc.

**Figure 9:**
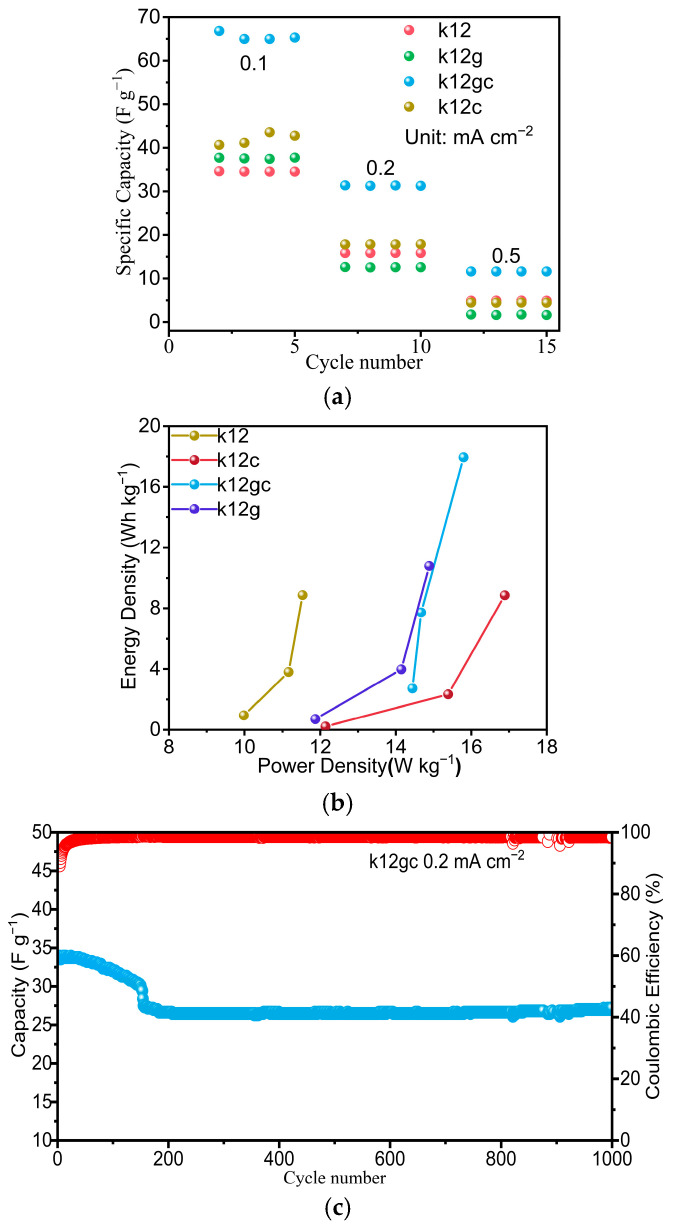
The test results of the electrochemical performance of each sample. (**a**) Rate plot of each sample; (**b**) Ragone plots of each sample; (**c**) cycling performance of k12gc at 0.2 mA cm^−2^. Solid symbols (left y-axis): capacitance retention; open symbols (right y-axis): Coulombic efficiency.

Figure 8 displays the galvanostatic charge–discharge (GCD) profiles. The k12gc sample exhibited the smallest voltage drop, 0.0083 V, consistent with the electrochemical impedance spectroscopy (EIS) results. This agreement indicates that coating the electrode with GO + CNTs markedly diminishes the capacitor’s voltage drop and increases the number of electrochemically active sites for charge storage. A comparative analysis of the GCD profiles further shows that the k12gc composite has the lowest IR drop, which is corroborated by its reduced semicircle diameter in the Nyquist plot. Figure 9a summarises the rate capability of each sample. At a current density of 0.1 mA cm^−2^ (normalised to the mass of NCB), the k12gc sample delivered the highest specific capacitance, 66.8 F g^−1^; when normalised to the total electrode mass, this corresponds to approximately 7.16 F g^−1^. This value is substantially higher than those of samples containing only one conductive additive or none. The electrochemical performances were further compared using a Ragone plot in Figure 9b. The k12gc composite attained superior energy–power characteristics, reaching a maximum energy density of 18.0 Wh kg^−1^ and a power density of 15.8 W kg^−1^ at 0.1 mA cm^−2^. These results validate the effectiveness of the GO/CNT co-modification strategy.

Table S1 situates our work among representative CSSC systems reported from 2023 to 2026. A common limitation in these studies is the trade-off between electrochemical performance and mechanical strength: achieving high capacitance generally requires increased carbon loading, which weakens the matrix. Our k12gc composite adopts an alternative strategy. At an exceptionally low total nanomaterial loading of 0.125 wt%, it achieves a 58.4% increase in effective conductivity, a 63% reduction in charge-transfer resistance and improvements in compressive and flexural strength of 4.8% and 8.3%, respectively. These concurrent gains arise from the complementary functions of CNTs, which provide electronic conduction, and GO, which templates the microstructure, distinguishing our approach from single-filler or electrolyte-centred strategies.

The cycling stability of the k12gc composite was assessed by galvanostatic charge–discharge over 1000 cycles at 0.2 mA cm^−2^ (Figure 9c). The CSSC showed an initial specific capacitance of 24.1 F g^−1^, which fell by about 38.7% during the first 200 cycles. After this decline, the capacitance entered a stabilisation phase with minimal further loss (<2% per 100 cycles) over the remaining 800 cycles. The Coulombic efficiency remained above 95% for the entire 1000-cycle period.

Capacitance retention and Coulombic efficiency describe fundamentally different aspects of cycling performance and are not inherently correlated. Coulombic efficiency measures the charge reversibility of an individual charge–discharge cycle; a value approaching 100% indicates that almost all the charge injected during charging is recovered on discharge, implying that parasitic side reactions (for example, electrolyte decomposition or irreversible Faradaic processes) are negligible. By contrast, capacitance retention quantifies the progressive loss of available charge storage capacity over extended cycling and is typically determined by extrinsic degradation mechanisms such as deterioration at the electrode–current collector interface, electrolyte dry-out from the pore network, or mechanical disruption of the conductive percolation network. Importantly, these degradation processes can reduce the absolute capacitance while leaving the remaining active material’s charge reversibility intact, so a high Coulombic efficiency can coexist with falling capacitance.

**Figure 10:**
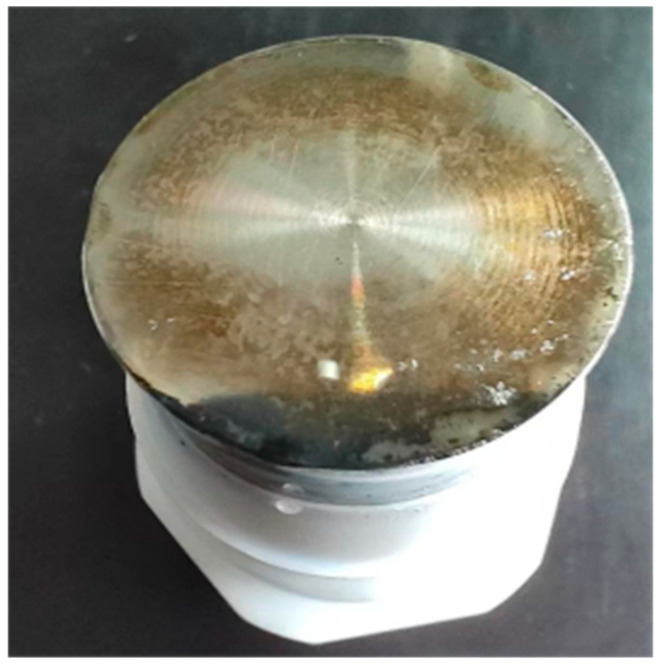
Photo of the stainless steel current collector after 200 cycles.

The consistently high Coulombic efficiency (>95%) suggests that the intrinsic charge-storage mechanism, specifically the formation of electric double-layer capacitors (EDLC) on the NCB/CNT carbon network, remains highly reversible throughout the cycling process. The pronounced initial capacitance loss (~38.7% within 200 cycles) is therefore chiefly attributable to degradation at the electrode–current collector interface, as evidenced by a black passivation layer formed on the stainless-steel current collector after cycling (Figure 10). This corrosion layer raises interfacial contact resistance and effectively isolates a portion of the electrode from the external circuit, reducing the measurable capacitance, while the electrochemical reversibility of charge storage at the remaining connected active sites is preserved. The subsequent capacitance stabilisation after ~200 cycles implies that a dynamic equilibrium is established at the corroded interface and that the GO-CNT reinforced composite matrix retains satisfactory intrinsic structural and electrochemical durability. Figure 9c has been revised to clearly distinguish the capacitance–retention curve (left y-axis, solid symbols) from the Coulombic-efficiency curve (right y-axis, open symbols), with explicit axis labels and a legend identifying each data series.

## 4. Conclusions

This study presents a rational functional-division strategy that uses trace amounts of CNTs and GO to overcome the conventional performance trade-off in CSSCs. By integrating one-dimensional CNTs (0.1 wt%) and two-dimensional GO (0.025 wt%) synergistically into a Portland cement matrix containing 12 wt% NCB, a hierarchical “electron–ion” conductive network is formed.

Electrochemically, CNTs form a three-dimensional percolation network that promotes efficient electron transport; this increases electrode conductivity by 58.4% and lowers charge-transfer resistance by 63%. Concurrently, GO acts as a template for a denser C-S-H gel microstructure, improving ionic pathways. Together, these effects produce a specific capacitance of 66.8 F g^−1^ at 0.1 mA cm^−2^ and enhance both energy and power density. Mechanically, CNTs bridge microcracks while GO refines the pore structure, decreasing harmful macropores (>200 nm) by 27.9%. This microstructural optimisation raises compressive strength by 4.8% and flexural strength by 8.3%.

In summary, the distinct but complementary roles of CNTs as electronic conduits and GO as ionic and structural templates constitute an effective design principle for high-performance, multifunctional CSSCs. Future work should adopt stable current collectors (for example, platinum) to limit interfacial corrosion and thereby improve long-term cycling stability.

## Figures and Tables

**Table 1:** Chemical composition and physical properties of ordinary silicate cement.

LOSS ^a^	SiO_2_	Al_2_O_3_	Fe_2_O_3_	CaO	MgO
3.31	24.99	8.26	4.03	51.42	3.71

^a^: LOSS: The organic and moisture content lost upon heating the cement to high temperatures (about 950 °C to 1000 °C).

**Table 2:** Mix proportions of cement-based electrodes.

Sample	Carbon/Cement	CNTs/Cement	GO/Cement	Water/Cement
k10	0.10	0	0	1.410
k10gc	0.10	0.001	0.00025	1.410
k12	0.12	0	0	1.825
k12c	0.12	0.001	0	1.825
k12g	0.12	0.001	0.00025	1.825
k12gc	0.12	0.001	0.00025	1.825

In the table, k represents NCB, and the following numbers indicate its mass ratio relative to cement (e.g., k12 = NCB/cement = 0.12). CNTs and GO contents are also expressed as mass ratios relative to cement; g denotes GO and c denotes CNTs.

## Data Availability

The original contributions presented in this study are included in the article/Supplementary Material. Further inquiries can be directed to the corresponding author.

## Supplementary Materials

The following supporting information can be downloaded at https://www.mdpi.com/article/10.3390/ma19102116/s1, Figure S1: Preparation of cement-based structural supercapacitor electrodes; Figure S2: Schematic diagram of CSSCs; Figure S3: Scanning Electron Microscopy (SEM) images (a) CNTs, (b) GO; Figure S4: CV curves of various samples at different scanning speeds (a) k12, (b) k12g, (c) k12c, (d) k12gc; Figure S5: CV curves of various samples at different scanning speeds (a) k10, (b) k10gc; Table S1. Comparison of electrical conductivity, specific capacitance, and mechanical properties with representative cement-based structural supercapacitors.
