# Supplementary material for: Effect of CNTs and GO Additives on Mechanical and Electrochemical Properties of Cement Structural Supercapacitors
Source: Materials (Basel). 2026 May 18;19(10):2116. doi: 10.3390/ma19102116 (PMC13208346; doi:10.3390/ma19102116)
Supplement: Supplementary file 1 [file materials-19-02116-s001.zip › materials-4274639-supplementary.pdf]

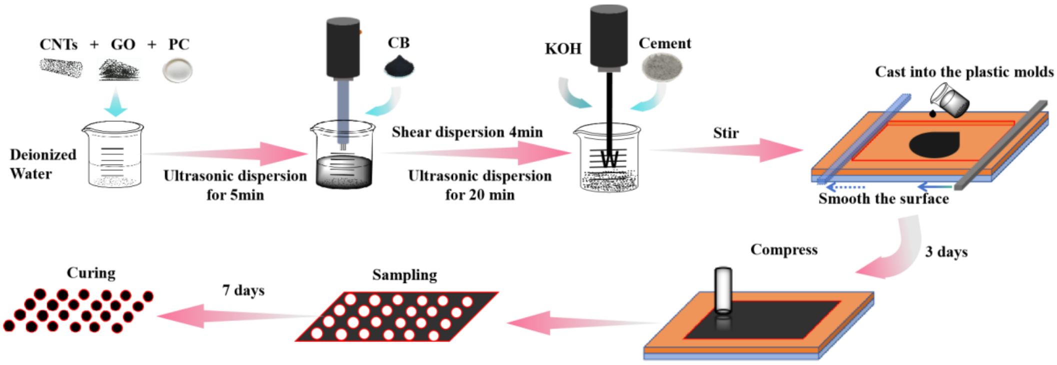

Figure S1 Preparation of cement-based structural supercapacitor electrodes

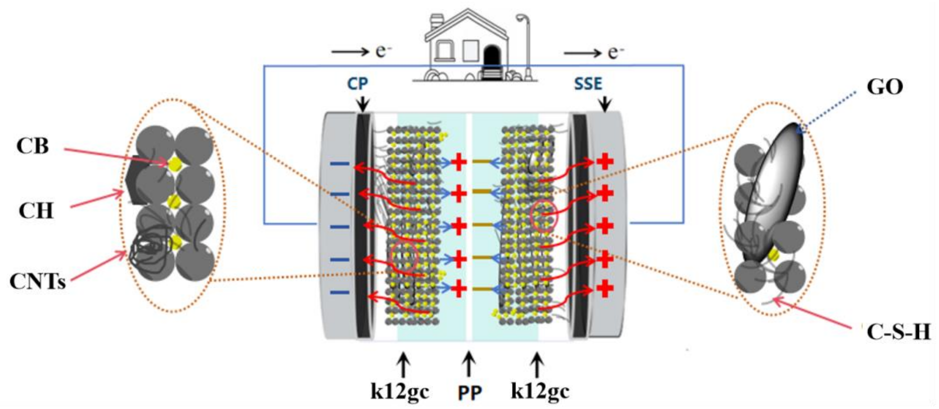

Figure S2 Schematic diagram of CSSCs

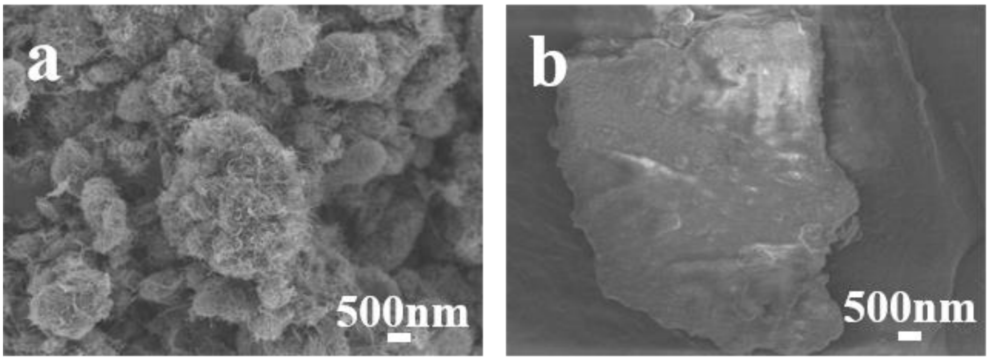

Figure S3 Scanning Electron Microscopy (SEM) images (a) CNTs, (b) GO

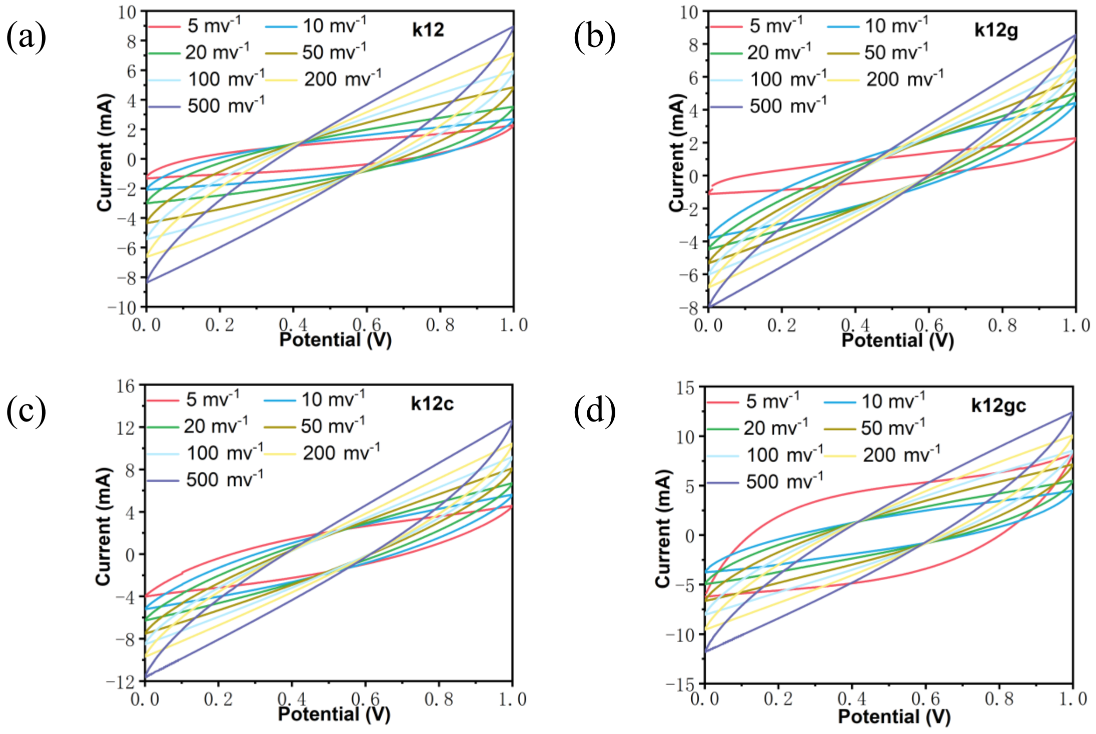

Figure S4 CV curves of various samples at different scanning speeds (a) k12, (b) k12g, (c) k12c, (d) k12gc

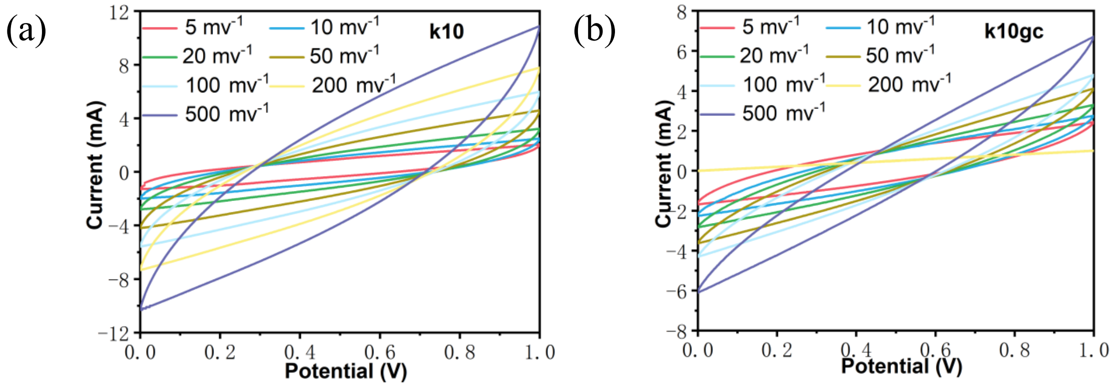

Fig. S5 CV curves of various samples at different scanning speeds (a) k10, (b) k10gc

Table S1. Comparison of electrical conductivity, specific capacitance, and mechanical properties with representative cement-based structural supercapacitors.

Ref.	Electrode/Electrolyte Strategy	Electrical Conductivity (S cm^{-1}) / Ionic Conductivity (mS cm^{-1})	Specific Capacitance	Compressive / Flexural Strength (MPa)
This work (CCE)	NCB 12% + CNTs 0.1% + GO 0.025%	σ_eff: 0.16 (58.4%↑ vs k12)	66.8 F g^{-1} (0.1 mA cm^{-2})	5.27 / 2.10 (+4.8% / +8.3% vs k12)
Chanut et al. (2023) (CCE)	nCB in cement	-	20–220 Wh m^{-3}	—
Yan et al. (2024) (CCE)	CB 40% in cement	-	2188 mF cm^{-2}	5.51 / —
Mao et al. (2025) (CCE)	CB 15% + CNT 1.5%	-	1562 mF cm^{-2}	23.1 / —
Li et al. (2025) (CCE)	nCB 5% + CF 2 vol%	-	217.88 mF cm^{-3}	36.88 / —
Liu et al. (2026) (CCE)	CB + PAM hydrogel (SDS)	Resistivity: 3 Ω·cm	1708 mF cm^{-2}	8.0 / —
Guo et al. (2025) (CCE mortar)	CNT-cement + rGO@Agg	Resistivity: 21.5 Ω·cm	350.0 mF cm^{-3}	32.22 / 9.72
Oumer et al. (2026) (CBE)	PPC + KFCN redox	-	52.67 mF cm^{-2}	16.67 / —
Zhao et al. (2026) (CBE)	Porous cement (PVA/SA) + CNT/Zn	-	560.4 mF cm^{-3}	21.9 / —
Shi et al. (2024) (CBE)	rGO/NF + porous cement ($KI+H_2O_2$)	-	351.5 mF cm^{-2}	18.1 / —
